# Sulfated Glycoaminoglycans and Proteoglycan Syndecan-4 Are Involved in Membrane Fixation of LL-37 and Its Pro-Migratory Effect in Breast Cancer Cells

**DOI:** 10.3390/biom9090481

**Published:** 2019-09-12

**Authors:** Chahrazed Habes, Günther Weber, Caroline Goupille

**Affiliations:** 1 INSERM, Université de Tours, UMR 1069 Nutrition, Growth and Cancer, 37032 Tours, France; chahrazed.habes@etu.univ-tours.fr (C.H.); caroline.goupille@univ-tours.fr (C.G.); 2 CHRU de Tours, Hôpital Bretonneau, 37032 Tours, France

**Keywords:** LL-37, breast cancer, glycosaminoglycans, syndecan, cell migration

## Abstract

Initially characterized by its antimicrobial activities, LL-37 has also been shown to significantly contribute to tumor development. On breast cancer cell lines, LL-37 increases intracellular calcium via the TRPV2 channel and their migration via the activation of PI3K/AKT signaling. Its all-d enantiomer d-LL-37 induces similar effects, which excludes a protein-protein interaction of LL-37 in a classic ligand-receptor manner. Its net charge of +6 gave rise to the hypothesis that the peptide uses the negative charges of sulfoglycans or sialic acids to facilitate its attachment to the cell membrane and to induce its activities. Whereas several vegetal lectins, specifically attaching to sialylated or sulfated structures, blocked the activities of LL-37 on both calcium increase and cell migration, several sialidases had no effect. However, the competitive use of free sulfated glycoaminoglycans (GAGs) as chrondroitin and heparin, or treatment of the cell surface with chondroitinase and heparinase resulted in an activity loss of 50–100% for LL-37. Concordant results were obtained by blocking the synthesis of GAGs with 4-Methylumbelliferyl-β-d-xyloside, and by suppression of glycan sulfatation by sodium chlorate. Using a candidate approach by suppressing proteoglycan synthesis using RNA interference, syndecan-4 was shown to be required for the activities of LL-37 and its binding to the cell surface. This leads to the conclusion that syndecan-4, by means of sulfated GAGs, could act as a receptor for LL-37.

## 1. Introduction

The LL-37 peptide is released from the C-terminus of the Human Cathelicidin Antimicrobial Protein hCAP18. Initially characterized by its antimicrobial activities, LL-37 is now considered as multifunctional, inducing chemotaxis, stimulating angiogenesis and promoting tissue repair [1,2]. Increasing evidence has demonstrated a significant role in a variety of cancer where, depending on the cancer origin, both pro- and antitumorigenic effects have been reported [3,4]. 

Mechanistically, these activities have been linked to the activation of multiple membrane-associated proteins, transmembrane receptors of different classes as well as ion channels [4,5]. Some of them have been suggested as being direct receptors for LL-37, although their physical interaction remains to be characterized. However, their variety and structural diversity did not suggest a conventional receptor-ligand binding of LL-37 to these proteins as a common denominator for their activation. In cell proliferation studies on lung cancer cell lines [6] and NIH3T3 fibroblasts [7], the all- d- enantiomer of LL-37 showed activities identical to those of the wildtype l- enantiomer. Likewise, the promigratory activities of LL-37, depending on AKT signaling and the activation of the TRPV2 Ca^2+^-channel, were induced by both l- and d-enantiomer [8]. Binding of LL-37 to the cell membrane has been shown to reduce the mobility of membrane receptors of dendritic cells and keratinocytes [9], and to decrease cell membrane fluidity of breast cancer cells [8]. This suggested that LL-37 may influence transmembrane receptors through indirect mechanisms of action, by binding to the membrane interface. 

Binding studies of LL-37 on bacterial membranes [10] and model micelles [11,12,13,14] have demonstrated that it adopts the conformation of an amphipathic helix, inserting its hydrophobic site within the hydrophobic/hydrophilic interface. Phospholipids increased binding of LL-37 [13], whereas cholesterol was shown to attenuate its attachment [14]. 

On the MDA-MB-435s cancer cell line, however, LL-37 was localized at the surface of pseudopodia and caveolae [8]. These structures are known to harbor receptors activated by LL-37 [15,16,17] but are rich in cholesterol, which should have prevented LL-37 from binding to the membrane. To explain this contradiction, we hypothesized that LL-37 needed to associate with nonlipid structures in addition to the lipid bilayer. 

Some scattered data have previously demonstrated that glycans may play a role in regulating the activities of LL-37. Its antimicrobial activities in the airway can be suppressed by binding to mucins, probably due to unspecific interactions between the positively charged peptide and the negatively charged glycans [18]. Similarly, its proinflammatory activities on epithelial cells and macrophages can be neutralized by a synthetic glycan [19]. An active role of proteoglycans in lipid rafts was demonstrated for the endocytosis of a LL-37-DNA complex [20] and the uptake of lipopolysaccharides mediated by LL-37 [21]. It thus appeared conceivable to investigate the impact of proteoglycans to the activities of LL-37 in cancer cells. 

We hypothesized that LL-37, due to its six positive charges, might interact with negatively charged glycans (sialic acid or sulfated glycans). We thus tried to identify glycans and glycoproteins able to transduce the LL-37 activities we have characterized previously, i.e., stimulation of constitutive calcium entry and cell migration on our mammary tumor cell lines.

## 2. Materials and Methods 

### 2.1. Cell Lines

MCF7, MDA-MB-435s and MDA-MB-231 cell lines were obtained from ATCC (via LGC Standards, Molsheim, France) and grown in Dulbecco’s modified Eagle’s medium (DMEM) containing 4.5 g/L glucose, supplemented with 10% fetal calf serum (FCS) (Eurobio, Courtaboeuf, France). Cells were grown at 37 °C in a humidity saturated atmosphere containing 5% CO_2_. The cells were reseeded 48 h prior to the experiments described below. 

### 2.2. Peptides, Inhibitors and Antibodies Used in this Study

The LL-37 peptide (Sequence: LLGDFFRKSK EKIGKEFKRI VQRIKDFLRN LVPRTES) and its enantiomer d-LL-37 were synthesized and HPLC-purified to >95% (GeneCust, Dudelange, Luxembourg, and GL Biochemicals, Shanghai, China). All experiments were performed at 10 μg/mL (2.2 μM) of peptide if not mentioned otherwise. Enzymes, inhibitors and antibodies and the concentration used in this study are listed in Supplementary Tables S1 and S2.

### 2.3. Cell Migration Assay

Twenty four hours before cell migration experiments cells were adapted to the migration medium, DMEM containing 0.1% FCS and 0.1% Bovine Serum Albumin (BSA). Cells (30,000 for MDA-MB-453s and MDA-MB-231, 60,000 for MCF7) suspended in 300 µL of migration medium at the upper side of a migration chamber (Insert with Polyethylene filter with 8 µM pores, Falcon BD Biosciences, Le Pont de Claix, France), together with the enzymes or inhibitors at the concentrations listed in Supplementary Table S1. Inhibition with 4-Methylumbelliferyl-β-d-xyloside and sodium chlorate required a pretreatment for 48h at 1 mM and 30 mM, respectively, before the migration experiment. The lower chamber was filled with 800 µL of migration medium containing or not LL-37. After 5 h for MDA-MB-453s and MDA-MB-231 and 24 h for MCF7, cells were fixed with methanol and stained with DAPI at 1 µg/mL, and counted in a fluorescent microscope using Image J (https://imagej.net). To highlight the potential inhibitory effects of reagents, results are normalized to migration of cells with LL-37. In all migration experiments, controls are performed using 5% FCS as attractant, to monitor unspecific effects of the reagents used in the study.

### 2.4. Measurements of Intracellular Cα^2+^ Variation

Intracellular Ca^2+^ concentration variations were detected using the fluorimetric assay with Fura2-AM (a ratiometric probe for intracellular Ca^2+^) as essentially described [8]. Intracellular Ca^2+^ variations were evaluated using the fluorescence (510 nm) emission ratio at excitations at 340 and 380 nm. This ratio is directly related to the amount of intracellular calcium. Briefly, measurement started when cells were suspended in buffer without Ca^2+^, for recording the basal fluorescence level. After 20 s, Ca^2+^ was applied at 2 mM leading to calcium influx through opened membrane calcium channels (Control calcium level). To evaluate LL-37 and the effects of inhibitors, they were added to the resuspended cells just before the beginning of the measurement. The fluorescence level obtained for cells in presence of 2 mM calcium and LL-37 was used as reference to normalize subsequent measurements. To calculate the effect of inhibitors to LL-37, the normalized Ca influx in presence of LL37 (100%) and the channel activity at 2 mM Ca and in absence of LL-37 (0%—indicated as a horizontal line in the graphs) are used as reference points.

### 2.5. Immunofluorescence Labeling

Forty eight hours before experiment, 20,000 cells were seeded in black plate with transparent flat well (96 wells, 10082471, Corning, Wiesbaden, Germany). The medium was replaced for 24 h with OPTI-MEM (Life Technologies, Saint Aubin, France) with 0.2% Bovine Serum Albumin (BSA, Sigma-Aldrich, St. Quentin Fallavier, France). Enzymes or inhibitors (for concentration see Supplementary Table S1) were applied in d-PBS with 1% BSA for 1 h. After two washes in d-PBS, LL-37 was incubated at 10 µg/mL for 10–15 min at 4 °C; then, cells were fixed with Paraformaldehyde 4% as described in [8]. For the detection of LL-37, a mouse monoclonal antibody [22] was used at 2 µg/mL.

### 2.6. RNA Interference and Expression Analysis by Quantitative Real-Time Polymerase Chain Reaction (qRT-PCR)

The siRNAs used in this study are listed in Supplementary Table S2. Cells were transfected in suspension using Lipofectamine RNAiMax (Fisher Scientific, Illkirch, France) according to the manufacturer’s protocol, at a final siRNA concentration of 25 nM. Cells were seeded in six-well plates at 250,000 per well. Experiments were then performed 72 h after transfection. While for all experiments on syndecan-1 and syndecan-4 published siRNA were used [23,24], an additional siRNA was designed for migration experiments on MDA-MB-435s to ascertain the specificity of the observations on syndecan-4 suppression. 

Transcript suppression was determined by qRT-PCR 72 h post transfection. For expression analysis, total RNA was extracted using the NucleoSpin^®^RNA II kit (Macherey-Nagel, Hoerdt, France), and reverse transcribed using the PrimeScript^TM^ RT Reagent Kit (Perfect Real Time, TAKARA, Nice, France). Gene quantification was performed in the LightCycle^®^ 480 II (ROCHE) thermocycler, with 20 ng of cDNA using the SYBR^®^ Premix Ex Taq^TM^ (Tli RNaseH Plus, TAKARA) kit at a final volume of 10 µL. Primers were used at a final concentration of 500 nM and are listed in Supplementary Table S2. After preincubation at 50 °C for 5 min and 95 °C for 10 min, 45 amplification cycles (95 °C 10 s, 60 °C 30 s, 72 °C 20 s) were performed. The expression was determined relative to HPRT1 as reference, and expressed using the ∆∆Ct method [25].

### 2.7. Statistic Analysis

The significance of the results was determined by Mann-Whitney non-parametric statistics. The results are expressed as means ± SEM. The significance was indicated by stars (* *p* < 0.05, ** *p* < 0.01, *** *p* < 0.001). The number N of independent measurements is indicated in the figure legends.

## 3. Results

### 3.1. The Activities of LL-37 Are Blocked by Lectins but Do Not Require α2–3- or α2–6-Linked Sialic Acids

Since we assumed that the activities of LL-37 on the cancer cell might be reduced by blocking glycans on the cell surface, our first strategy was to mask negatively charge glycans such as sialic acid using lectins.

**Figure 1:**
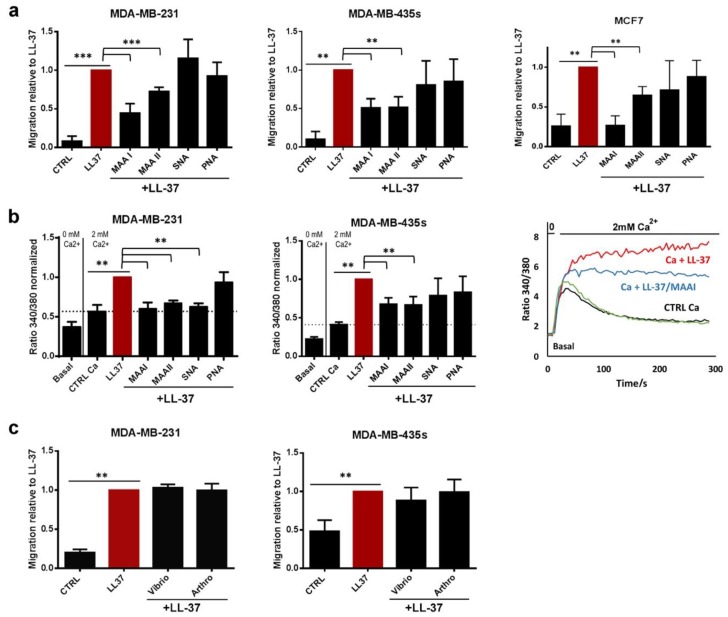
LL-37-induced migration and calcium entry is suppressed by lectins but not by removal of sialic acids. (**a**) Migration of MDA-MB-231, MDA-MB-435s and MCF7 induced by LL-37 (10 µg/mL) with or without lectins (5 µg/mL), MAA I and MAA II (*Maackia amurensis* I and II), SNA (*Sambucus nigra* Agglutinin) and PNA (Peanut Agglutinin) (N = 8, 6 or 3). (**b**) Calcium entry in MDA-MB-231 and MDA-MB-435s (N = 4) at conditions as in (**a**). To the right, a display of the time course of fura-2 fluorescence ratio detected at 510 nm with both excitations at 340 and 380 nm is shown. The graph to the right shows representative curves for the time course of the fura-2 fluorescence ratio at 510 nm with excitations at 340 and 380 nm. (**c**) Migration of MDA-MB-231 and MDA-MB-435s induced by LL-37 (10 µg/mL) after treatment with sialidases of *Arthrobacter ureafaciens* or *Vibrio cholerae* (treatment at 0.1 UI/mL for 1 h) that preferentially digested α2–6 andα2–3 sialic acids, respectively (N = 4). Data (migration and calcium entry) were normalized to the effect of LL-37. Statistics are relative to control without LL-37, with *** *p* < 0.001, ** *p* < 0.01, * *p* < 0.05, and relative to the effect by LL-37.

Four lectins, *Maackia amurensis* Agglutinin I and II (MAA I, MAA II, α2–3 sialic acid specificity), lectin *Sambucus nigra* Agglutinin (SNA, α2–6 sialic acid specificity) and an irrelevant lectin Peanut Agglutinin (PNA, galactose specificity) were assayed during cell migration, which we initially used as a reporter experiment for the activities of LL-37. The α2–3 or α2–6-linked sialic acids were markedly present on MDA-MB-231 and MDA-MB-435s as shown in Supplementary Figure S1. However, since glycosylation patterns vary in cancer tissues and cell lines and depend on their origin and malignancy [26,27,28], three cell lines, MDA-MB-435s, MDA-MB-231 and MCF7, were compared in the experiments. In all cell lines, only lectins MAA I and MAA II, which bind terminal α2–3-linked sialic acid [29] significantly reduced cell migration (Figure 1a), whereas SNA and PNA showed no suppressive effect. The level of suppression varied among the lines: in presence of MAA I and II, migration of MDA-MB-231was suppressed by 50% and 30%, respectively, by 50% for both lectins for MDA-MB-435s, and 100% and 40%, respectively, for MCF7. With the exception of MAA I on MDA-MB-435s, lectins did not suppress cell migration in control experiments, in which 5% FCS was used as chemoattractant (not shown). 

We have previously shown that the promigratory activity of LL-37 in breast cancer cell lines is linked to the activation of the TRPV2 Ca-channel [8] and influx of extracellular calcium. As demonstrated in Figure 1b, this activity was almost totally abrogated in presence of lectins MAA I, MAA II and SNA for MDA-MB-231 and significantly reduced by 45% in MDA-MB-435s with both lectins MAA I and MAA II. The basic influx of extracellular calcium remained unaltered in the presence of lectins alone (Figure 1b). An apparent difference was observed on the treatment with the SNA lectin, which induced increased migration despite decrease of calcium. However, control experiments with 5% FCS as chemoattractant revealed that this was due to an unspecific promigratory effect of SNA (Supplementary Figure S1a).

Throughout this article calcium measurements in MCF7 were not presented since calcium influx were too small to report reliable inhibitions. However, TRPV2 calcium influx implication in LL-37 activities in MCF7 has been previously demonstrated [8].

To gauge whether α2–3- or α2–6-linked sialic acids were implicated in the activities of LL-37, two sialidases from *Arthrobacter ureafaciens* and *Vibrio cholerae* [30] were used to remove sialic acids from plasma membranes. Enzyme efficacies were performed in a control experiment performed in parallel, verifying a decrease of binding MAA lectins (Supplementary Figure S2b,c) and LL-37 peptide (Supplementary Figure S2d) on the cell plasma membrane. Removing sialic acids from membranes of MDA-MB-231 and MDA-MB-435s did not alter the promigratory effect of LL-37 (Figure 1c). Taken together, these results suggested that sialic acids contributed to LL-37 fixation on the plasma membrane but were not involved in the promigratory activities LL-37.

### 3.2. Membrane Chondroitin Sulfate and/or Heparin Are Involved in Membrane Binding of LL-37 and Are Needed for Its Activities

**Figure 2:**
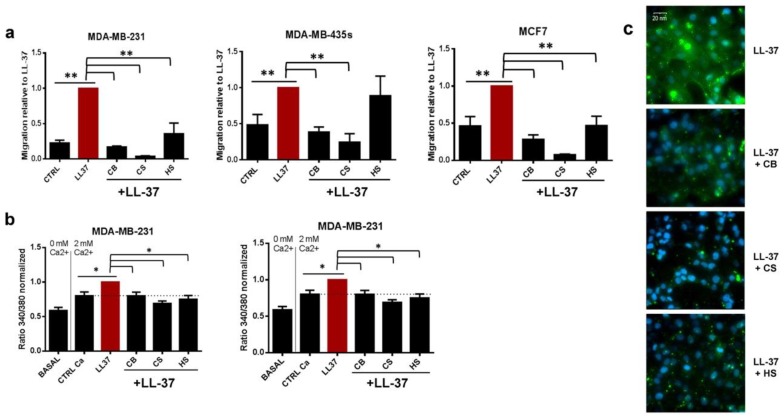
Glycoaminoglycans (chondroitin sulfate and heparin) inhibit LL-37 induced-calcium entry and migration and reduce its attachment to the cell surface. (**a**). Migration of MDA-MB-231, MDA-MB-435s and MCF7 cell lines induced by LL-37 and incubated or not with different glycoaminoglycans (CB—chondroitin sulfate B at 0.5 mg/mL, CS—chondroitin sulfate from shark cartilage at 0.5 mg/mL, HS—heparin at 50 UI/mL (N = 4)). (**b**) Calcium entry in MDA-MB-231 and MDA-MB-435s induced by LL-37 (10 µg/mL) with or without glycoaminoglycans with concentrations as used for migration (N = 3). (**c**) Immunofluorescence labeling with anti-LL-37 [22] plus secondary antibody (Alexa488-green) on MDA-MB-231, incubated with or without different glycoaminoglycans with same concentrations as used for migration. Nuclei were labeled by DAPI (in blue). Magnification 400×. Data (migration and calcium entry) are normalized to LL-37. Statistics: ** *p* < 0.01, * *p* < 0.05, relative to LL-37 and relative to control without LL-37.

Since some previous studies have demonstrated an interaction between sulfated oligosaccharides of mammalian cells and LL-37 [21,31], the first experiments were performed using different free sulfated glycoaminoglycans such as chondroitin and heparin. In the three cell lines, both chondroitins and heparin totally abrogated LL-37 effects on migration (Figure 2a) or calcium influx (Figure 2b), except for heparin on MDA-MB-435s migration. Control migration experiments with 5% FCS, as chemoattractant revealed that this inhibitions was not due to decrease of migratory capacities of the cells (Supplementary Figure S1b). Moreover, the inhibition was linked to a reduced binding of LL-37 to the cell surface as shown an immunofluorescence analysis performed on nonpermeabilized MDA-MB-231 cells (Figure 2c). As previously reported, LL-37 attached to the cells in a heterogenous manner, reflecting its accumulation at specific sites of the cell surface [8]. As shown in Figure 2c, membrane fixation of LL-37 was strongly reduced by chondroitins and heparin (Figure 2c).

**Figure 3:**
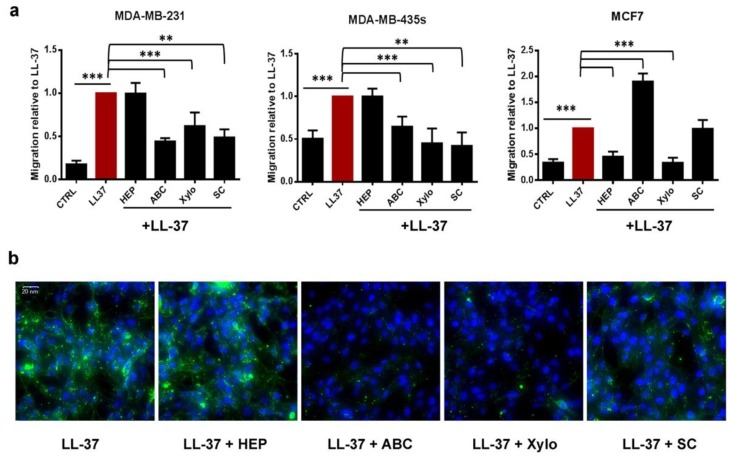
Digestion of membrane chondroitin sulfate and heparin and inhibition of glycoaminoglycan synthesis decrease membrane fixation of LL-37 and cell migration. (**a**) Migration of MDA-MB-231, MDA-MB-435s and MCF7 cell lines induced by LL-37. Cells were previously incubated or not with different enzymes digesting glycoaminoglycans (Hep: heparinases I/III, treatment 5mUI/mL 1 h, ABC: chondroitinase ABC, treatment 1 UI/mL 1 h, Xylo: 4-Methylumbelliferyl-β-d-xyloside 0.5 mM) or with an inhibitor of sulfatation (SC—sodium chlorate 30 mM) (N = 4). (**b**) Immunofluorescence labeling with anti-LL-37 on MDA-MB-231 cells previously treated or not as in (a). Magnification ×400. Data are normalized to LL-37. Statistics: *** *p* < 0.001, ** *p* < 0.01, * *p* < 0.05, relative to LL-37 and relative to control without LL-37, respectively.

To ascertain that glycoaminoglycans did not act only as competitors but were biological actors in LL-37 activities, several strategies were elaborated to reduce or modify glycoaminoglycans on the cell membranes. The first one involved enzymatic digestion of chondroitins by chondroitinase ABC and heparin or heparan by heparinase I/III. Whereas chondroitinases ABC inhibited LL-37-induced migration by 65% and 53% in MDA-MB-231 and MDA-MB-435s, respectively, no effect of this enzyme was observed in MCF7 cells (Figure 3a). Inversely, heparinases presented no inhibitory effect in LL-37-induced migration on MDA-MB-231 and MDA-MB-435s but abrogated 80% of LL-37-induced migration in MCF7 (Figure 3a). The migration inhibition was specific on LL-37 since in corresponding experiments, using FCS as attractant, no inhibition or even a slight increase was observed (Supplementary Figure S1b). In correspondence, fluorescence microscopy revealed that on MDA-MB-231 only chondroitinases were able to suppress its binding to the cell membrane, while heparinase showed no effect (Figure 3b).

The second strategy was to block glycosylation synthesis of proteoglycans using a modified xylose (4-Methylumbelliferyl-β-d-xyloside) as xylose is the first and necessary glycan to connect glycoaminoglycans on proteoglycans [32]. In the three cell lines, xyloside strongly reduced LL-37-induced migration by 45%, 82% and 100% in MDA-MB-231, MDA-MB-435s and MCF7, respectively (Figure 3a). Regarding LL-37-induced calcium influx, xyloside inhibited by 30% and 50% calcium influx in MDA-MB-231 and MDA-MB-435s.

Finally, the last strategy was to suppress the sulfatation of glycoaminoglycans using sodium chlorate [33]. Except for MCF7, in which sodium chlorate did not affect LL-37-induced migration, pretreatment with NaClO_3_ led to a decrease by 60% and 86% of migration of MDA-MB-231 and MDA-MB-435s (Figure 3a). Concordantly, calcium influx was decreased by 80% and 50% in these cell lines (Supplementary Figure S3). 

Taken together, these results suggested that LL-37 needed membrane glycoaminoglycans to exercise its activities. Their nature, however, appears to differ among the cell lines, as chondroitins seemed to be more implicated for MDA-MB-231 and MDA-MB-435s cells and heparin/heparan for MCF7. The effects were specific to LL-37 since none of these treatments (glycoaminoglycans digestion, synthesis blockage or sulfatation inhibition) interfered with cell migration capabilities when 5% FCS was used as chemoattractant (Supplementary Figure S1b). 

### 3.3. Identification of Syndecan-4 as Proteoglycan Implicated in Membrane Fixation and LL-37-Induced Activities

**Figure 4:**
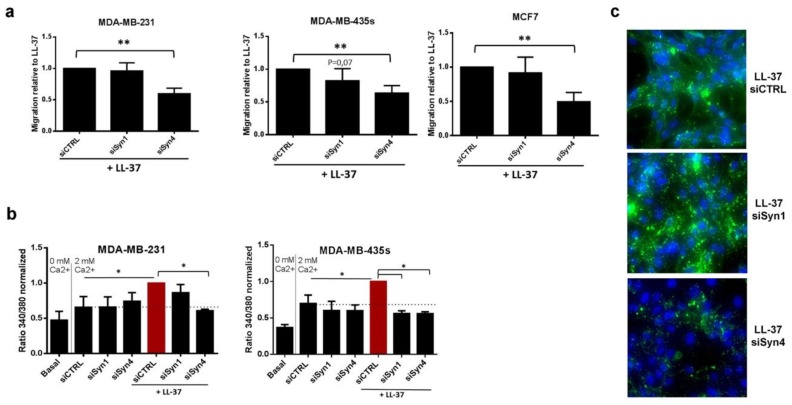
Syndecan-4 is implicated in LL-37 induced-calcium entry and cell migration. Effects of RNA interference against syndecan-1 and 4 on (**a**) LL-37-induced migration of MDA-MB-231, MDA-MB-435s and MCF7 (N = 5). (**b**) Calcium entry in MDA-MB-231, MDA-MB-435s cell lines induced by LL-37 (N = 3). (**c**) Immunofluorescence labeling with anti-LL-37 (Alexa488-green) on MDA-MB-231, treated with siRNA against syndecans-1 and 4. Nuclei labeled by DAPI (in blue). Magnification 400x. Data are normalized to the effects of LL-37. Statistics: ** *p* < 0.01, * *p* < 0.05.

Growing evidence has shown that proteoglycans (PGs) and glycoaminoglycans (GAGs) are essential partners during tumor progression [34]. Among PGs, syndecan-1 and -4 are associated with breast cancer [35,36] and cell adhesion and mobility [37,38] and were, therefore, selected as candidates for the activities of LL-37. RNA interference against syndecan-4 (the efficacy of RNAi for both genes presented in Supplementary Figure S4) reduced LL-37-induced migration by 40–50% in the three cell lines. No reduced migration was observed when targeting syndecan-1 (Figure 4a) in MCF7 and MDA-MB-231, and a non-significant tendency to reduction in MDA-MB-435s. Control experiments with FCS as chemoattractant confirmed that the reduced migration was specific for treatment with LL-37 (Supplementary Figure S1c).

LL-37-induced calcium influx was entirely abolished in MDA-MB-435s when targeting syndecan-1 or syndecan-4. In MDA-MB-231, suppression of syndecan-1 resulted in less decrease of calcium influx than suppression of syndecan-4 (Figure 4b). The immunofluorescence analysis revealed that binding LL-37 to the surface of MDA-MB-231 was strongly reduced after RNA interference against syndecan-4. In contrast suppression of syndecan-1 rather appeared to increase LL-37 binding. (Figure 4c). We conclude that the attachment of LL-37 to syndecan-4 is a common denominator for mediating its activities in all experiments performed. 

In addition, the impact of syndecan-4 on cell migration was assayed on MDA-MB-435s using the d-enantiomer of LL-37. RNA interference against syndecan-4 (completed with the use of a second siRNA) identically suppressed the promigratory activities of both enantiomers in an identical manner (Supplementary Figure S5). 

## 4. Discussion

Our investigation has revealed that surface glycans are required for the promigratory activities of LL-37. The concentration of LL-37, as used in this and our previous studies, is well within the physiological concentrations observed in vivo [39,40], being highly elevated in breast cancer [41]. Stimulatory effects at this concentration have been published on growth and/or migration of primary epithelial cells and cell lines [8,41,42], while cytotoxic effects were observed at concentrations higher than used in this study [39], (GW, data not shown). It thus appears reasonable to assume that the data presented here are in agreement with physiological conditions. 

The electrostatic interaction between sialic acids and cationic antimicrobial peptides has been demonstrated before [43]. In fact, the removal of both sialic acids and sulfated glycans decreased the binding of LL-37 to the cell surface, which may reflect these unselective electrostatic interactions. However, only sulfated structures contribute to the activities of LL-37 we assayed. Among these, O-sulfatation appears involved since the inhibition by sodium chlorate selectively affects O-sulfotransferases [33]. In conclusion, the activities of LL-37 require its binding to rather specific glycan structures.

In a candidate approach, we have identified syndecan-4 as a proteoglycan that critically mediates the activities of LL-37. Its localization, binding properties and activities may help to explain a number of previous findings of the behavior of LL-37. Similar to LL-37, syndecan-4 induces cell migration [44] and is known to cluster at lipid rafts prior to its endocytosis [45], which agrees with our localization studies for LL-37 [8]. Moreover, syndecan-4 can activate various signaling pathways, both by binding various growth factors [46] and by directly interacting with tyrosine kinase receptors as a co-receptor [47,48]. The fact that the d- and l- enantiomer of LL-37 shows identical activities has been attributed to its interaction with the lipid bilayer of the cellular membrane [6,7,8]. In our present study, however, both enantiomers lost their activity after suppression of syndecan-4, suggesting that both bind equally to the glycan structures of syndecan-4. Such behavior has previously been reported for both enantiomers of a small cell-penetrating peptide, the cellular uptake of which require binding to the heparan sulfate structures of syndecan-4 [49]. 

In the light of these findings, we hypothesize that syndecan-4, via its GAGs moieties, serves as a primary target for mediating the activities of LL-37. This does certainly not contradict previous findings of its attachment to the cellular membrane—glycans may actually serve as a “guide” for LL-37 to support its attachment to lipid rafts that otherwise would have been avoided by the peptide, as suggested by the experiments on artificial membranes [14].

MAA I and MAA II lectins, which inhibited the activities of LL-37, are best characterized as binding to terminal α2–3 sialic acid linked to galactose. Binding of MAA II has been also reported to sulfated galactose [29,50], but, to our knowledge, not to chondroitin sulfate or heparan sulfate. These lectins thus do not appear to directly compete with LL-37 on its binding site, and therefore, the implication of sialylated structures for LL-37 activities requires deeper investigations in our models.

The responsible glycan structures involved in the activities of LL-37 appear to vary, since heparinase abolished LL-37-induced migration in MCF7, whereas chondroitinases and sodium chlorate were more efficient on MDA-MB-231 and MD1-MB-435s. Chondroitin sulfates have been reported as more abundant and more expressed in MDA-MB-231 as compared to MCF7 [51]. For breast cancer in general, alterations in the expression of glycan modifiers and sulfatation patterns has been correlated to the metastatic potential of the tumor [52], thus, our findings might reflect these differences. 

Heparan Sulfate Proteoglycans have been suggested as mediating the immunomodulatory effects of LL-37 [53] although their nature remained unknown. Our discovery of syndecan-4 as a major player for LL-37 does not exclude that other proteoglycans are involved as well. This notion is supported for example on MCF7 cells where heparinase and xyloside inhibited its activities more effectively than RNA interference against syndecan-4. We also noted that the suppression of syndecan-1 appeared to modify—rather increase—binding of LL-37 to the cell surface of MDA-MB-231—certainly without increasing the activities of LL-37. In absence of evidence, it might be hypothesized that syndecan-1 modifies the access of the peptide to the cell surface, but without involving the locations where the peptide induces cell migration and calcium influx (i.e., caveolae and pseudopodia [8]). Irrespective of some differences in response of the cell lines to inhibitors of cell surface glycan structures, syndecan-4 appears to be a common denominator for the activities of LL-37 we have assayed thus far. 

LL-37 is rapidly internalized [8], an aspect it shares with small cell penetrating peptides. Although these peptides are structurally unrelated to LL-37, their positive charge could be seen as a denominator in common. Their internalization has been shown to depend on their association with glucosaminoglycans [54,55], or more specifically with syndecans [56]. These findings may provide some insight to the affinities of LL-37. 

## 5. Conclusions

Changes in glycosylation and the remodeling of glycans are part of cancer development [57], and the expression of some of these antigens and their modifying enzymes has been associated with the metastatic nature of the tumor [52] and poor prognosis [58]. Proteoglycans in particular and signaling pathways emerging from them contribute to the motility of breast cancer cells and thus their metastatic potential [34]. Our results indicate that LL-37 could be an integral part of their activities.

## Supplementary Materials

The following are available online at https://www.mdpi.com/2218-273X/9/9/481/s1.
