# Supplementary material for: Sulfated Glycoaminoglycans and Proteoglycan Syndecan-4 Are Involved in Membrane Fixation of LL-37 and Its Pro-Migratory Effect in Breast Cancer Cells
Source: Biomolecules. 2019 Sep 12;9(9):481. doi: 10.3390/biom9090481 (PMC6769752; doi:10.3390/biom9090481)
Supplement: Supplementary file 1 [file biomolecules-09-00481-s001.pdf]

Supplementary Materials

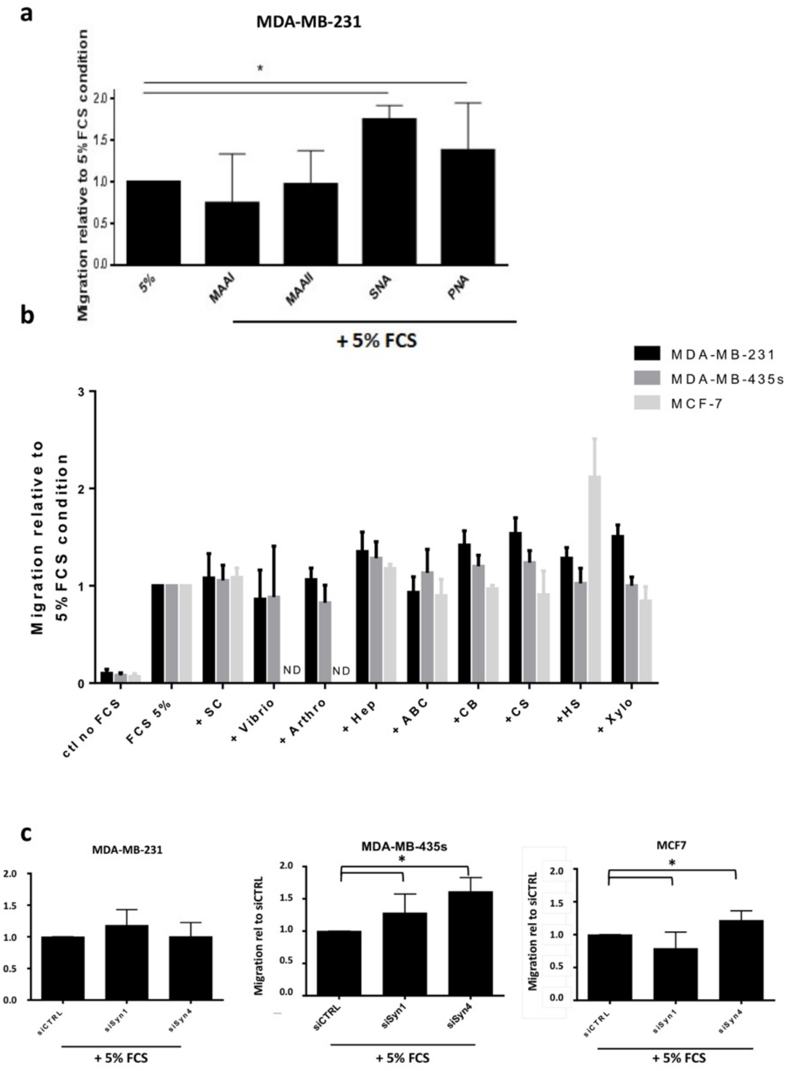

Suppl Figure S1: **Experimental control for the specificity of the cellular migration experiments**. As a chemoattractant 5% FCS was used instead of LL-37. (**a**) Control experiment for the use of lectins on MDA-MB-231 , revealing unspecific migration stimulation SNA and PNA; **(b)** controls for the use of competing glycans and glycan degrading enzymes; **(c)** for the suppression of syndecans-1 and -4 by RNAi. (N≥3), *p<0.05.

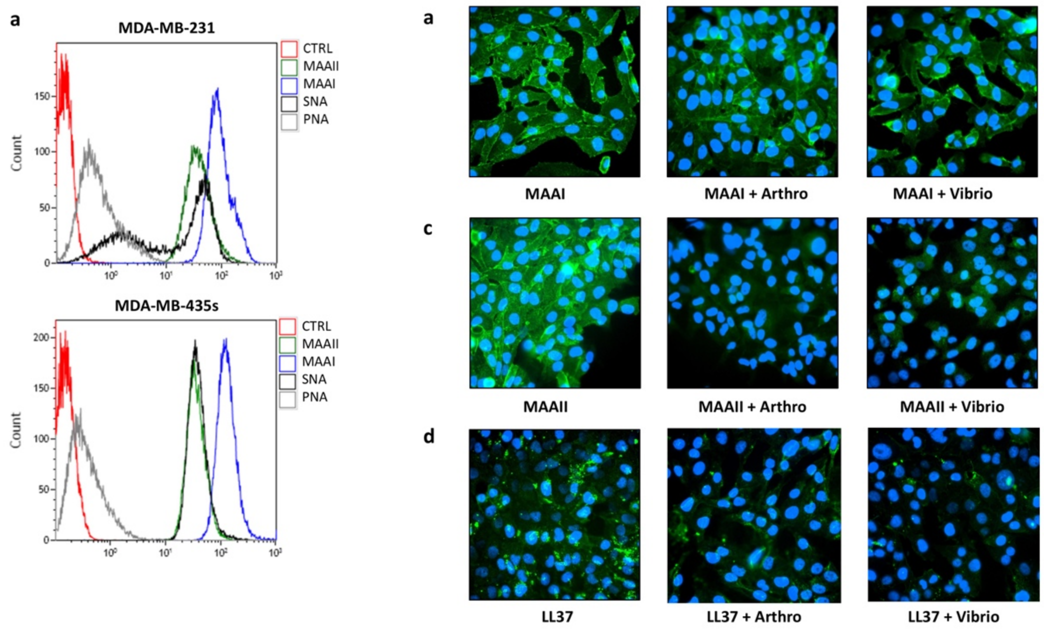

Suppl Figure S2: **The α2–3 or α2–6-linked sialic acids are involved in fixation of LL-37 on the cellular membrane.** **(a)** The α2–3 or α2–6-linked sialic acids are markedly present on MDA-MB-231 and MDA-MB-435s. Cells incubated with or without biotinylated lectins (5μg/mL), MAA I and MAA II (*Maackia amurensis I and II)* lectins which preferentially recognize α2–3 sialic acids, SNA *(Sambucus nigra* Agglutinin) lectin which preferentially recognize terminal α2–6 sialic acids or PNA (Peanut Agglutinin*)* lectin which bind at terminal Gal β1-3GalNAc. After labelling with streptavidin-Alexa488, fluorescence was analyzed by flow cytometry. **(b),(c)** The sialidases of *Arthrobacter ureafaciens* and *Vibrio cholerae* (preferentially digested α2–6 and α2–3 sialic acids, respectively) decrease MAA I and MAA II fixation on membranes of MDA-MB-231 cells**.** Nuclei were labelled by DAPI (in blue). **(d)** The α2–3 or α2–6 sialic acids are involved in LL-37 fixation on cellular membrane. Immunofluorescence labelling LL-37 (Alexa 488- green color) on MDA-MB-231 cells that were previously treated or not with sialidases of *Arthrobacter ureafaciens* or *Vibrio cholerae* that preferentially digested α2–6 and α2–3 sialic acids, respectively. Nuclei were labelled by DAPI (in blue). Mgnification x400.

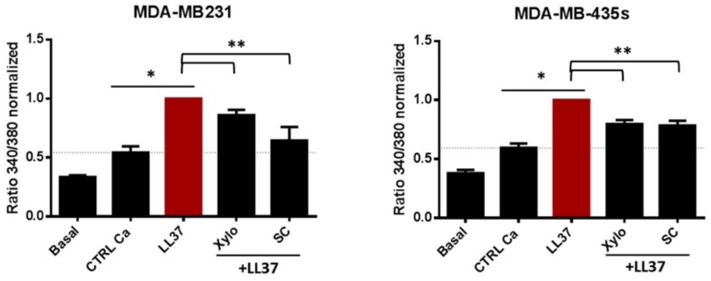

Suppl Figure S3: **The sulfatation and the glycoaminoglycans linked to proteoglycans via xylose are involved in LL-37 induced- calcium entry.** The MDA-MB-231 and MDA-MB-435s breast cancer cells were previously incubated or not with 4-Methylumbelliferyl-β-D-xyloside (Xylo) or with an inhibitor of sulfatation (SC-sodium chlorate) before analysis for the calcium entry. Data are normalized to LL-37. Statistics: **p<0.01, *p<0.05, relative to LL-37 (N≥3) and relative to control without LL-37.

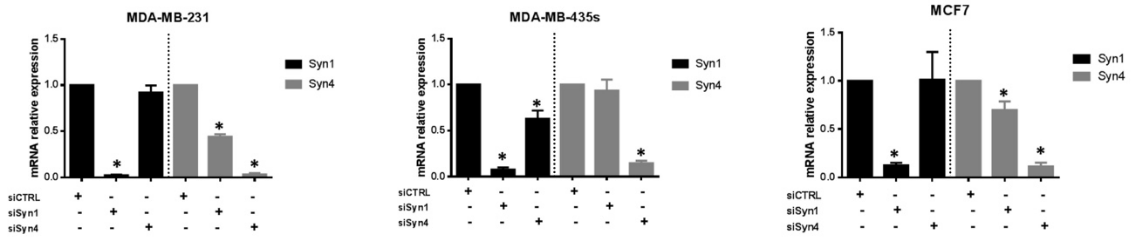

Suppl Figure S4: **Efficacy of siRNA for syndecan-1 and syndecan-4 in breast cancer cells lines.** The MDA-MB-231, MDA-MB-435s and MCF7 breast cancer cells were transfected with control siRNA (siCTRL), for syndecan-1 (siSyn1) or syndecan-4 (siSyn4) and mRNA expression was evaluated by q-PCR 72h after transfection (N≥3). The expression levels are presented relative to the respective control condition using of expression with siCTRL.

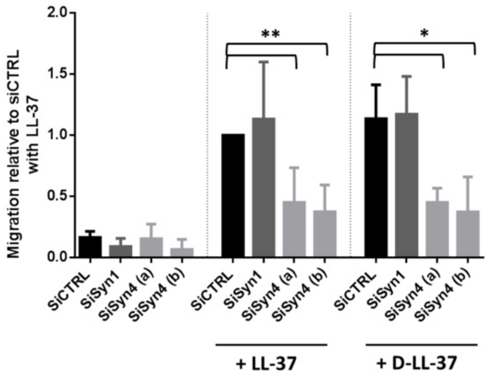

Suppl Figure S5: **RNA interference against syndecan-4 equally suppress the promigratory activities of both L- and D- enantiomer of LL-37.** For SDC4, two siRNAs were used against different target sites of the transcripts in migration experiments to verify the specificity of our observation. Suffixes (a) and (b) refer to sequences in Table S2 . Cell migration performed on MDA-MB-435s as above, **d**ata normalized to the effect of LL-37. Statistics: **p<0.01 *p<0.05, N≥4

Table S1: **Products, suppliers and concentrations used in this study**

	**Products and activity preferences**	**Suppliers and reference number**	**Concentration of use**
Enzymes	Neuraminidase *Vibrio cholerae*<br>Digests sialic acids α2–3>α2–6 or α2–8	Roche Diagnostics (Mannheim Germany)<br>11 080 725 001	0,1 UI/mL<br>In PBS + 0.01% BSA 1h
	Neuraminidase *Arthrobacter ureafaciens*<br>Digests Sialic acids α2–6 ≥ α2–3 > α2–8	Roche Diagnostics<br>10 269 611 001	
	Chondroitinase ABC (*Proteus vulgaris*)<br>Digests Hylauronic acid, Chondroitin sulfate, Dermatan Sulfate	Sigma-Aldrich<br>C3667	1 UI/mL in PBS + 0.01% BSA 1h
	Heparinases I and-III blend (*Flavobacterium heparinum*) Degrade heparin, heparan sulfate and S-domains of heparan sulfate	Sigma-Aldrich<br>H3917	5 mUI/mL Tris-HCl 20 mM, 100 mM NaCl, 1,5 mM CaCl2, pH 7,5 + 0.01% BSA 1h
Antibodies	Mouse monoclonal anti LL-37	Ref [22]	2 µg/ml
	Streptavidin-DyLight 488	Vector laboratories<br>SA-5488	1/2000
	secondary antibody anti mouse-Alexa488	Invitrogen<br>A11001	1/2000
Inhibitors	Lectin *Maackia amurensis* Agglutinin (MAA) Recognized Sialic acids α2–3<br>MAA I<br>MAA II	Vector laboratories, (Peterborough, United Kingdom) B-1315 & B-1265	5 µg/ml
	Lectin *Sambucus nigra* Agglutinin (SNA) Recognized Sialic acids α2–6	Vector labs B-1305	
	Lectin Peanut Agglutinin (PNA)<br>Recognizes Galβ3GalNAc	Vector labs BA-0074	
	Chondroitin sulfate sodium from Shark	Sigma-Aldrich C4384	0,5 mg/ml
	Chondroitin B	Sigma-Aldrich C3788	0,5 mg/ml
	Heparin sodium salt	Sigma-Aldrich H3149	50 UI/mL
	4-Methylumbelliferyl-β-D-xyloside	Sigma-Aldrich M7008	0,5 mM
	Sodium chlorate	Sigma-Aldrich<br>1064201000	30 mM

Table S2: **Sequences for primers and siRNA used in this study**

Genes	Primer Forward 5'-3'	Primers Reverse 5'-3'
HPRT1	TGACCTTGATTTATTTTGCATACC	CGAGCAAGACGTTCAGTCCT
SDC1 (Syndecan-1)	AGGATGGAGGTCCTTCTGC	CCGAGGTTTCAAAGGTGAAGT
SDC4 (Syndecan-4)	CCTCAGTTGCACTAACCACG	AGCTGAGGCTGTGACTCGTT
**SiRNA**	**siRNA seq target**	**Reference/Suppliers**
Control	-	Qiagen, Cat No./ID: 1027310
Syndecan1	AGGACUUCACCUUUGAAACC	Ref [23]
Syndecan4 (a)	AAGGCCGAUACUUCUCCGGAG	Ref [24]
Syndecan4 (b)	CAUCGUGGGCAUCCUCUUUGCCG	Eurogentech
